# Congenital Cytomegalovirus-Related Hearing Loss

**DOI:** 10.3390/audiolres14030043

**Published:** 2024-06-16

**Authors:** Nicoleta Gana, Iulia Huluță, Mihai-Ștefan Cătănescu, Livia-Mihaela Apostol, Florina Mihaela Nedelea, Romina-Marina Sima, Radu Botezatu, Anca Maria Panaitescu, Nicolae Gică

**Affiliations:** 1 Filantropia Clinical Hospital, 011171 Bucharest, Romania; gana_nicoleta@yahoo.com (N.G.); iuliahuluta16@gmail.com (I.H.); stefan.catanescu0920@stud.umfcd.ro (M.-Ș.C.); romina.sima@umfcd.ro (R.-M.S.); radu.botezatu@umfcd.ro (R.B.); anca.panaitescu@umfcd.ro (A.M.P.); gica.nicolae@umfcd.ro (N.G.); 2 Gynecology Department, Faculty of Medicine, Carol Davila University of Medicine and Pharmacy, 020021 Bucharest, Romania; livia-mihaela.cosma@rez.umfcd.ro

**Keywords:** hearing loss, CMV infection, congenital infections, screening

## Abstract

Congenital hearing loss is a significant global health concern that affects millions of newborns and infants worldwide, posing substantial challenges for affected individuals, their families, and healthcare systems. This condition, present at birth, can stem from genetic factors, in utero exposures, infections, or complications during pregnancy or childbirth. The spectrum of congenital hearing loss ranges from mild to profound, impacting the development of speech, language, and cognitive skills, thereby influencing educational achievements, social integration, and future employment opportunities. Early detection and intervention strategies, such as newborn hearing screenings, genetic counseling, and the use of hearing aids or cochlear implants, are crucial for mitigating these impacts. This review article aims to explore the diagnostic approaches and management strategies for congenital cytomegalovirus-related hearing loss, emphasizing the importance of interdisciplinary care and the potential for technological advances to improve outcomes for affected individuals.

## 1. Introduction

Since the turn of the 21st century, newborn hearing screening programs have been implemented across North America, Europe, and most developed nations. These programs, which screen all newborns for hearing loss rather than just those considered at risk, have led to the identification of permanent bilateral hearing loss at a rate of approximately 1.33 per 1000 live births [1]. The incidence of severe to profound bilateral hearing loss in newborns remains steady at 1.1 per 1000, while an additional 1 to 2 per 1000 newborns exhibit mild to moderate bilateral hearing loss or unilateral hearing loss of varying degrees. Despite these consistent prevalence rates, the age at diagnosis has significantly decreased, thanks to effective screening initiatives [2].

The etiology of congenital hearing loss can be divided into genetic causes and acquired conditions [1]. Overall, 80% of congenital hearing loss is caused by genetic conditions. They classify as syndromic (10%) or non-syndromic (90%), the latter being autosomal recessive, autosomal dominant, or X-linked transmitted [3]. The acquired conditions refer to congenital infections such as CMV (cytomegalovirus), Zika virus, Rubella, and intrauterine exposure to toxins [1,4].

The general occurrence of hearing loss in congenital CMV infection (cCMV) is 12.6% [5,6,7]. cCMV ranks as the most prevalent congenital infection globally, with reported birth prevalence rates ranging from 0.64% to 0.7% [5,6,7]. Symptomatic children face a higher risk, with one in three experiencing hearing loss, compared with one in ten asymptomatic children [5,6,7]. When these findings are applied to the broader population, it suggests that out of every 10,000 children born annually, 5 will develop hearing loss due to cCMV [5,6,7]. Morton et al. approximated that cCMV is implicated in 21% of hearing loss cases at birth and contributes to 25% of childhood hearing loss by the age of 4 [8]. The exact mechanisms leading to sensorineural hearing loss from congenital CMV infection remain largely unknown [1]. Besides the direct damage caused by the virus, there is also evidence of immune system damage triggered by the host’s immune reaction and the virus’s pro-inflammatory chemokines [9,10]. The severity of the infection and the immune response from the mother, fetus, and placenta play a vital role in the health outcomes of the infection [9,10]. At birth, about 10% of newborns with CMV are symptomatic, with the highest risk of neonatal symptoms occurring when the mother is infected around the time of conception or during the first trimester [9,10].

## 2. Materials and Methods

**Figure 1:**
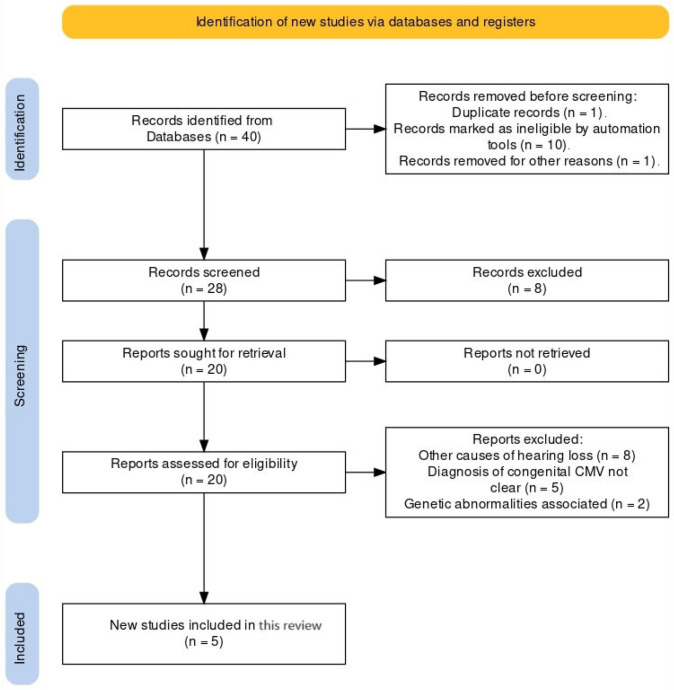
PRISMA flow diagram.

Under the Preferred Reporting Items for Systematic Reviews and Meta-Analyses (PRISMA) criteria, a systematic search was conducted across scientific literature databases including PubMed, Science Direct, and Google Scholar to identify relevant studies published up to 2024 on congenital cytomegalovirus (CMV) and hearing loss (Figure 1). Only studies focusing on congenital CMV-related hearing impairment were included, while articles that did not meet the defined diagnosis criteria and addressed different causes of hearing loss were excluded.

## 3. CMV Infection during Pregnancy

CMV, belonging to the human herpesvirus family, is the predominant viral reason for congenital infections. It stands as the primary non-genetic reason behind sensorineural hearing loss (SNHL) and is a significant source of neurological disorders [11]. Table 1 summarizes the findings of recent studies on the association between CMV infection during pregnancy and SNHL in neonates. Approximately 10–15% of newborns with congenital CMV exhibit symptoms at birth, with up to 25% of these children facing long-term health issues [12].

**Figure 2:**
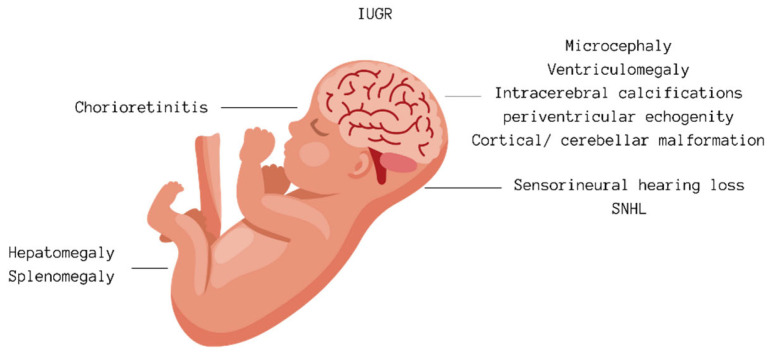
Fetal abnormalities potentially indicative of CMV infection [11,14,15,16,17,18,19].

CMV infection during pregnancy can either be a primary infection or result from the reactivation of a previous infection or infection with another strain of the virus. The virus is transmitted to the fetus through the placenta, with the likelihood of transmission being higher during a primary infection in pregnancy than with a non-primary infection. The risk of congenital CMV infection is roughly 30–40% for infants whose mothers have a primary infection during pregnancy, compared with a 1–2% risk for infants following a non-primary maternal infection [7,13]. The risk of congenital infection also changes depending on when during the pregnancy the primary infection occurs, rising from about 30% in the first trimester to 47% in the third trimester [14,15,16,17,18,19]. Although viral transmission risk is lower early in pregnancy, the risk of diagnosing severe fetal infection prenatally is greater if the infection happens in the first rather than the third trimester. Consequently, the adverse outcomes for the fetus tend to be more serious, particularly with infections in the first trimester [14,15,16,17,18,19] (in Figure 2). Most women experiencing a primary CMV infection during pregnancy do not show symptoms, but a small number may exhibit symptoms similar to infectious mononucleosis, such as fever, fatigue, muscle pain, swollen lymph nodes, and, in rarer cases, hepatitis and pneumonia, with few experiencing lasting effects. Like other herpesviruses, CMV can stay dormant in the body, especially in the salivary glands, and can reactivate at any time, including during pregnancy [1,2].

Diagnosing maternal CMV infection involves identifying primary infection either by the emergence of CMV-specific IgG in previously seronegative women or through the detection of CMV IgM antibodies alongside low IgG avidity. However, it is challenging to rule out non-primary maternal infections using serological tests. Because of the absence of effective treatments during pregnancy and the failure to meet several criteria for an effective screening test, routine antenatal CMV screening is not commonly recommended [20]. Thus, serological testing for CMV is typically reserved for pregnant women presenting with flu-like symptoms, signs of glandular fever not attributed to Epstein–Barr virus, hepatitis symptoms with negative tests for hepatitis A, B, and C, or when routine ultrasounds suggest fetal abnormalities potentially indicative of CMV infection [20]. These include intracranial calcifications, ventriculomegaly, and microcephaly, which may signal neurological implications, while hepatomegaly and polyhydramnios may denote hepatic and amniotic fluid abnormalities, respectively. Additionally, intrauterine growth restriction (IUGR) and echogenic bowel patterns might suggest compromised fetal development and gastrointestinal issues linked with CMV. Detection of echogenic foci in the heart chambers and hyperechogenicity in the kidneys further aids in recognizing cardiac and renal complications associated with congenital CMV infection [11] A woman’s immunity before conception does not ensure the protection of the fetus from infection [21].

**Table 1:** Prevalence of hearing impairment in children with CMV.

Study	Conclusion
Lanzieri et al., 2017 [22]	At age 18, SNHL prevalence was 25% among case patients and 8% among controls. The risk of delayed-onset SNHL was not significantly greater for case patients than for controls. For case patients, the risk of delayed-onset SNHL was significantly greater among those with unilateral congenital/early-onset hearing loss than those without. The prevalence of severe to profound bilateral SNHL among case patients was 2%.
Palma et al., 2019 [23]	Urinary CMV testing was carried out in 2966 children, representing 3.9% of total live births, between 2004 and 2014. CMV infection was confirmed in 339 children, and information on hearing loss was available in 250 (73.8%), of which 45/250 were cCMV, while 205/250 were acquired. A few children (n = 6/250-13%) with cCMV infection had confirmed hearing impairment. Among them, two were diagnosed after 2012 through the neonatal hearing screening program and were positive. The prevalence of symptomatic cCMV after the introduction of newborn hearing screening (2/10) was 20%, while the proportion of symptomatic cCMV with hearing loss before the screening was 11%. Among the 205 children (82%) with acquired CMV, 6 (2.9%) had moderate to severe hearing impairment. The remaining three cases were attributed to a delayed diagnosis of cCMV. All six cases with acquired CMV were born before the implementation of the newborn hearing screening.
Forner et al., 2014 [24]	The study aimed to analyze the kinetics of CMV viremia and viruria clearance in postnatal life after primary CMV intrauterine infection. All of the 33 newborns included were born full-term. Ten of thirty-tree infants (30%) developed postnatal sequelae during the first months or years of life. Eight children developed unilateral or bilateral hearing loss, where four infants developed severe bilateral hearing loss (average tone loss, 71–90 dB hearing level) four children presented moderate unilateral hearing loss (average tone loss, 41–70 dB hearing level). Two children developed psychomotor retardation, and one baby developed progressive right-side hemiparesis. The time of appearance of clinical abnormalities ranged from 3 months to 5 years of age. The remaining 23 infants (70%) presented no symptoms when the follow-up was concluded at 6 years of age.
Bradford et al., 2015 [25]	In the study, among 50 infants with serum samples, 37 tested positive for CMV DNA at enrollment, indicating viremia. These viremic infants were more prone to developing hearing loss at both the initial assessment and the 6-month follow-up. Additionally, they displayed other markers of active CMV disease, including elevated alanine aminotransferase levels, petechial rash, and organomegaly.
Picone et al., 2018 [15]	Following a retrospective analysis of 238 patients with maternal primary CMV infection identified during routine screening, the cohort underwent monitoring with serial ultrasound scans. The rate of intrauterine transmission was 24.9%, varying across different pregnancy periods. Maternal infections during the preconception or periconceptional period and the first trimester were associated with a significantly higher risk of ultrasound abnormalities compared with later periods. Among the infected newborns, three were symptomatic, all previously flagged during prenatal ultrasounds. Interestingly, no symptomatic fetal infections were observed when maternal infection occurred after the 14th week of gestation. Overall, 5.5% of clinically asymptomatic cases later developed hearing loss.

Lanzieri et al. defined sensorineural hearing loss as a hearing level of ≥25 dB for the ABR click or at any frequency for the corrected tone-burst or pure-tone air conduction results. This definition enabled the identification and categorization of sensorineural hearing loss in children with congenital cytomegalovirus infection. The audiometric configuration of hearing loss due to congenital CMV infection is not specific, and its severity varies. Children with congenital CMV infection may experience unilateral or bilateral hearing losses, ranging from unilateral high-frequency losses (4–8 kHz frequencies only) to profound bilateral losses. Progression and fluctuations in SNHL have been observed in these children as well. Additionally, there is a significantly higher risk of delayed-onset SNHL in children with unilateral congenital/early-onset hearing loss compared with those without it [22].

cCMV infection is primarily confirmed through PCR analysis by detecting CMV DNA in amniotic fluid, which is recommended to be conducted no sooner than 8 weeks following the estimated time of maternal infection and after 20 weeks of gestation. This timing is crucial as it aligns with established fetal urination, which affects the virus’s appearance in amniotic fluid. The sensitivity of amniotic fluid PCR for diagnosing fetal infection is influenced by the timing of amniocentesis relative to the maternal infection and the gestational age at which the procedure is performed [11].

The diagnosis of newborn hearing loss. Two screening tests have been validated by international consensus conferences for several years including otoacoustic emissions (OAEs) and automated auditory brainstem response (AABR). Both tests are designed to be administered by non-specialized personnel and yield a binary outcome as pass or fail. Each test has its advantages and disadvantages [26].

Otoacoustic emissions (OAEs) are sounds emitted from the outer hair cells of the cochlea in response to a sound stimulus calibrated at 35 decibels (dB) for screening purposes and are collected in the external auditory canal. OAE tests assess the entire auditory pathway from the outer ear to the inner ear [26,27]. The test is conducted 24 h post-birth during the infant’s natural sleep by placing a probe in the ear canal and recording from one ear to the other [26]. The rate of false positives, where OAEs are not detected despite no deafness, ranges from 3 to 8% on the first test and between 0.7 and 6% on a retest after 48 h; known causes include residual fluid in the outer or middle ear, movement, or excessive ambient noise [26,27]. Depending on the presence of risk factors, the rate of false negatives, where OAEs are detected in a deaf newborn, is between 0.009 and 0.2% [26]. This can occur in cases of auditory neuropathy due to damage to the inner hair cells of the cochlea (the actual sensory cells that transmit sound information to the auditory nerve fibers), auditory synaptopathy, or damage to the auditory pathways [26].

Automated auditory brainstem response (AABR) tests record the electrical activity of the auditory pathways up to the brainstem in response to a sound stimulus exploring frequencies between 2000 and 4000 Hz, with the device calibrated to deliver sounds at an intensity of 35 dB [26]. The test is performed on a naturally sleeping child, stimulating both ears simultaneously (with two disposable earphones attached to each ear) and recording the electrical activity with electrodes placed on the child’s forehead and nape. The rate of false positives (no electrical activity recorded despite no deafness) is between 0.2 and 0.8% after a retest. False negatives (normal AABR despite deafness) are limited to hearing losses affecting frequencies of 1000 Hz or lower [26,27,28,29].

A crucial factor in choosing between these techniques is that only AABR can detect deafness due to damage to the inner hair cells, the auditory nerve, or the auditory pathways in the brainstem. For newborns at risk (e.g., in level 3 maternity units, neonatology, and intensive care departments), hearing loss due to auditory pathway damage is much more common (about 10% of detected hearing losses) than in the general new-born population (1% of hearing losses), with varied causes like hyperbilirubinemia, anoxia, and neurological conditions or syndromes. Therefore, AABR is the preferred method for screening at-risk newborns [26].

Screening is typically conducted during the maternity hospital stay; a practice adopted by nearly all countries to ensure comprehensive coverage [26]. A targeted CMV approach testing newborns who fail hearing screening is useful to identify most children with CMV-related sensorineural hearing loss as the impact of environmental and genetic factors on viral infection rates is still not fully understood [21]. Unfortunately, many newborns and children affected by cCMV infection do not exhibit hearing loss at birth and later go on to have delayed onset hearing loss [21].

A case-controlled retrospective study performed on 35 adult patients with moderate to profound sensorineural hearing loss showed that cervical vestibular evoked myogenic potentials (VEMPs) are useful in increasing the diagnosis of underlying causes of SNHL [30].

In the newborn period, the diagnosis of congenital CMV (cCMV) is often made by detecting high levels of the virus in the infant’s saliva and urine. Finding viruses or viral DNA in these samples confirms cCMV infection. Testing urine or saliva within the first 2–3 weeks of life is crucial to differentiate cCMV from infections acquired at birth or through breastfeeding. While postnatal CMV infections can cause severe symptoms in very premature infants or those with significant immune deficiencies, they typically do not lead to long-term issues like sensorineural hearing loss (SNHL). Saliva testing for CMV using PCR technology is both highly accurate and reliable. To prevent contamination in breast milk, saliva samples should be collected at least an hour and a half after the last breastfeeding. Additionally, the integrity of PCR results is generally unaffected by the conditions under which samples are stored and transported [31,32].

Approximately 10% of infants infected with congenital CMV (cCMV) show symptoms at birth, including an enlarged liver and spleen, rashes such as petechiae or purpura, jaundice with elevated conjugated bilirubin levels, and/or a smaller head size (microcephaly). The consequences of cCMV infections vary widely; a significant number of symptomatic infants experience complications like sensorineural hearing loss (SNHL), cerebral palsy, delays in neurodevelopment, and vision loss [32]. Around half of these symptomatic newborns go on to develop SNHL, and among these, two-thirds exhibit neurological impairments. In contrast, 10–15% of infants who are asymptomatic but have SNHL will face permanent effects. For symptomatic infants, factors like intrauterine growth restriction and petechiae are linked to a higher risk of hearing loss, indicating a need for more research to pinpoint which children with asymptomatic cCMV are at risk for hearing loss.

SNHL associated with CMV can emerge later, progress over time, and vary in severity among children, regardless of whether they showed symptoms initially. About 50% of asymptomatic children with hearing loss have impairment in both ears. Because of the lack of visible symptoms at birth and the potential for SNHL to develop or worsen after birth, many infants with cCMV are not diagnosed through newborn hearing screenings [31]. Since it is challenging to predict which children, especially those who are asymptomatic, will develop SNHL, regular monitoring for hearing loss is advised for all children with cCMV every six months for the first 5–6 years. Early detection and timely intervention can significantly improve speech and language outcomes for children affected by CMV-related hearing loss. Both initial and subsequent CMV infections in the mother can lead to symptomatic cCMV infections and SNHL in the infant. While bilateral hearing loss, affecting speech development, occurs in nearly half of these infants, recent findings also highlight the negative impact of unilateral SNHL on a child’s overall development [31].

## 4. Advanced Diagnostic Approaches for Congenital Cytomegalovirus Infection

CMV DNAemia. Forner et al. investigated viral loads in the blood at birth as prognostic indicators for asymptomatic newborns after primary CMV infection during pregnancy. Significant differences in viral loads were found between infants developing late-onset diseases and those remaining asymptomatic. A threshold (≥12,000 copies/mL) indicated a high risk of CMV-related sequelae. Another threshold (≥17,000 copies/mL) was defined for predicting sensorineural hearing loss. These thresholds can aid early identification of at-risk infants, allowing for careful monitoring with audiological and neurodevelopmental assessments [33].

Amniotic fluid peptide biomarkers. A study analyzing the peptidome in CMV-infected amniotic fluids identified a prognostic classifier of 34 peptides, outperforming current fetal biomarkers in predicting asymptomatic or symptomatic status at birth. Emphasizing the significance of viral replication intensity, this classifier was associated with a subset of highly up-regulated proteins within a neurological disease network. Hence, this peptidome classifier could find application in clinical settings during pregnancy to enhance prenatal counseling [34].

Dried blood spot screening. One study presented an improved method for detecting CMV DNA in dried blood spots (DBSs) using a novel single-tube nested protocol, outperforming single-round PCR. DBS samples, already collected for newborn screening, provide a feasible avenue for CMV screening in high throughput settings. The research demonstrated an overall sensitivity of 81%, emphasizing the importance of assay methodology in newborn screening for CMV. The findings suggest the potential inclusion of CMV screening in newborn programs, enabling early identification and intervention for infected infants [35].

Apparent diffusion coefficient values of white matter in magnetic resonance imaging of the neonatal brain. Vande Walle et al. investigated the correlation between white matter apparent diffusion coefficient (ADC) values and clinical outcomes in newborns with cCMV. Elevated ADC values were significantly associated with neonatal hearing loss, cognitive impairment, and motor impairment during follow-up, serving as a predictive tool with an area under the curve ranging from 0.73 to 0.89. The findings suggest that white matter signal alterations are part of the symptomatic spectrum of cCMV. While the study has limitations, including small sample sizes and short follow-up periods, it highlights the potential of white matter ADC as a promising tool for predicting clinical outcomes in newborns with cCMV infection. Further research with larger cohorts and extended follow-up is needed to refine predictive models and establish clear cutoff values for clinical applications [36].

## 5. Congenital CMV Infection Treatment

To date, the possibility of treating fetal CMV infections with antiviral medication has been explored, particularly with Valaciclovir, showing potential benefits. A study assessed the effectiveness of administering a high dosage of oral valacyclovir, 8 g daily, to pregnant women whose fetuses were symptomatic with cytomegalovirus infection, focusing on those at high risk for neurosensory and neurological issues. The study was structured as a multicentric, open-label, phase II trial with a single arm. The findings suggest that when given at a high dosage during pregnancy, valacyclovir can positively impact the health outcomes of fetuses showing moderate symptoms of infection [32].

However, the use of more potent anti-CMV medications during pregnancy remains controversial because of concerns about their safety. Drugs like (val)ganciclovir, cidofovir, and foscarnet are not approved for pregnant women because of potential maternal and fetal risks. In contrast, valacyclovir, though less potent against CMV in vitro, has been deemed safer based on its track record of not increasing birth defect risks in the children of many women who used it during pregnancy. These medications work by blocking the virus’s DNA polymerase, thus preventing viral replication [36,37]. New drugs like Letermovir (LMV) and Maribavir (MBV) have been introduced as specific and effective anti-CMV options that operate through novel mechanisms, potentially reducing the side effects and toxicities associated with older drugs [38].

Congenital CMV infection is likely under-recognized because of a lack of adequate information on its prevention, the absence of universal guidelines for maternal and/or neonatal screening, and strategies for management and treatment. This situation hampers health policies aimed at mitigating the impact of this infection. Currently, CMV screening at birth is not universally implemented. Implementing a routine CMV screening for all newborns, including those without symptoms, could enhance their prospects. Universal screening has the potential to lower healthcare costs across the population. Additionally, early intervention could markedly improve the language development of these children, thus enhancing their social skills and learning abilities [9].

On the other hand, for newborns with cCMV infection, the two primary medications utilized are intravenous ganciclovir, administered at 6 mg/kg twice daily, and oral valganciclovir, which is a prodrug of ganciclovir. Upon oral intake, valganciclovir is quickly converted into ganciclovir. When valganciclovir is given at a dose of 16 mg/kg twice daily, the achieved plasma concentration is comparable to that of ganciclovir at a 6 mg/kg dosage [36]. Valganciclovir leads to a temporary reduction in the viral load, which reverts to its original level upon cessation of the treatment. Extended use of the drug could lead to the emergence of resistant viral strains. However, randomized controlled clinical trials have demonstrated that a 6-week course of treatment with ganciclovir and valganciclovir can be effective in newborns with symptomatic CMV infection affecting the central nervous system. This regimen has been associated with a decreased likelihood of severe hearing loss and improved neurological outcomes after one year [22,23].

A diagnosis of hearing loss early in a child’s life can lead to early intervention during the critical time of speech development. The goal of intervention is to improve the development of language, social skills, and literacy for children who are deaf or hard of hearing. Newborn hearing should be assessed no later than 1 month after birth, preferably before discharge from the birth hospital. Newborns who do not pass the initial hearing assessment should be given a comprehensive audiological examination at no later than 3 months of age and should receive intervention before 6 months of age [20,21].

Regarding congenital sensorineural hearing loss treatment, the universal screening of congenital hearing loss is recommended, as an early diagnosis promotes an early intervention to reduce the irreversible damage to speech development [38]. The treatment of congenital hearing loss depends on the underlying cause. When the cause is not known, an MRI should be performed to assess the anatomy of the internal ear. The first medication administered is oral corticosteroids. If no improvement is noted in 10–14 days, clinicians use salvage intratympanic steroids [39]. Hearing aids are used in chronic conditions. Surgical management is represented by cochlear implants in patients where hearing aids are not useful [40].

The evidence highlights a concerning lack of awareness among women regarding congenital cytomegalovirus (cCMV) infection when compared with other less prevalent congenital conditions such as Down syndrome, toxoplasmosis, and human immunodeficiency virus (HIV) infection. Insufficient awareness of cCMV infection is not limited to pregnant women alone; a mere fraction of healthcare providers currently address this issue with their patients. Recognizing this substantial gap, urgent action is warranted to implement health campaigns and sustained medical education initiatives. These efforts aim to raise awareness and enhance the standard of antenatal counseling, ultimately reducing the risk of cCMV transmission.

On the other hand, timely identification is crucial for prompt intervention, including providing auditory aids and specialized speech–language therapy before the age of 6 months, as recommended by the Joint Committee on Infant Hearing. This proactive approach significantly improves outcomes, facilitating smoother integration into mainstream education for affected children. While conducting clinical trials in pregnant women poses challenges, prioritizing preclinical studies is essential to advance our understanding and management strategies for cCMV infection.

## 6. Conclusions

This review sheds light on the significant impact of congenital cytomegalovirus (CMV) infection on newborn hearing loss, emphasizing its global health implications. By delving into the epidemiology, causes, diagnostic methods, management approaches, and recent advancements, our understanding of CMV-related hearing impairment was deepened. This review underscores the crucial role of early detection and intervention, particularly through newborn hearing screening programs, in promptly addressing hearing deficits. Additionally, it highlights the diverse clinical presentations of congenital CMV infection, underscoring the need for interdisciplinary collaboration and ongoing research endeavors.

## Data Availability

No new data were created or analyzed in this study. Data sharing is not applicable to this article.
